# Association of Genetic Risk Factors for Psychiatric Disorders and Traits of These Disorders in a Swedish Population Twin Sample

**DOI:** 10.1001/jamapsychiatry.2018.3652

**Published:** 2018-12-19

**Authors:** Mark J. Taylor, Joanna Martin, Yi Lu, Isabell Brikell, Sebastian Lundström, Henrik Larsson, Paul Lichtenstein

**Affiliations:** 1 Department of Medical Epidemiology and Biostatistics, Karolinska Institutet, Stockholm, Sweden; 2 MRC Centre for Neuropsychiatric Genetics and Genomics, Cardiff University, Cardiff, United Kingdom; 3 Institute of Neuroscience and Physiology, Gillberg Neuropsychiatry Centre, Centre for Ethics Law and Mental Health, University of Gothenburg, Gothenburg, Sweden; 4 School of Medical Sciences, Örebro University, Örebro, Sweden

## Abstract

**Importance:**

Psychiatric traits associated with categorically defined psychiatric disorders are heritable and present to varying degrees in the general population. It is commonly assumed that diagnoses represent the extreme end of continuously distributed traits in the population, but this assumption has yet to be robustly tested for many psychiatric phenotypes.

**Objective:**

To assess whether genetic risk factors associated with psychiatric disorders are also associated with continuous variation in milder population traits.

**Design, Setting, and Participants:**

This study combined a novel twin analytic approach with polygenic risk score (PRS) analyses in a large population-based twin sample.

Phenotypic and genetic data were available from the Child and Adolescent Twin Study in Sweden. Inpatient data were available for January 1, 1987, to December 31, 2014, and outpatient data for January 1, 2001, to December 31, 2013. The last day of follow-up was December 31, 2014. Data analysis was performed from January 1, 2017, to September 30, 2017.

**Main Outcomes and Measures:**

Questionnaires that assessed traits of autism spectrum disorder (ASD), attention-deficit/hyperactivity disorder (ADHD), learning difficulties, tic disorders (TDs), obsessive-compulsive disorder (OCD), anxiety, major depressive disorder (MDD), mania, and psychotic experiences were administered to a large Swedish twin sample. Individuals with clinical psychiatric diagnoses were identified using the Swedish National Patient Register. Joint categorical/continuous twin modeling was used to estimate genetic correlations between psychiatric diagnoses and continuous traits. The PRSs for psychiatric disorders were calculated based on independent discovery genetic data. The association between PRSs for each disorder and associated continuous traits was tested.

**Results:**

Phenotype data were available for 13 923 twin pairs (35.1% opposite sex and 31.7% same-sex females) at 9 years of age, 5165 pairs (36.9% opposite sex and 34.0% same-sex females) at 15 years of age, and 4273 pairs (36.5% opposite sex and 34.4% same-sex females) at 18 years of age. Genetic data were available for 13 412 individuals (50.2% females). Twin genetic correlations between numerous psychiatric diagnoses and corresponding traits ranged from 0.31 to 0.69. Disorder PRSs were associated with related population traits for ASD (β [SE] = 0.04 [0.01] at 9 years of age), ADHD (β [SE] = 0.27 [0.03] at 9 years of age), TDs (β [SE] = 0.02 [0.004] at 9 years of age), OCD (β [SE] = 0.13 [0.05] at 18 years of age), anxiety (β [SE] = 0.18 [0.08] at 9 years of age; β [SE] = 0.07 [0.02] at 15 years of age; and β [SE] = 0.40 [0.17] at 18 years of age), MDD (β [SE] = 0.10 [0.03] at 9 years of age; β [SE] = 0.11 [0.02] at 15 years of age; and β [SE] = 0.41 [0.10] at 18 years of age), and schizophrenia (β [SE] = 0.02 [0.01] at 18 years of age). Polygenic risk scores for depressive symptoms were associated with MDD diagnoses (odds ratio, 1.16; 95% CI, 1.02-1.32).

**Conclusions and Relevance:**

These results suggest that genetic factors associated with psychiatric disorders are also associated with milder variation in characteristic traits throughout the general population for many psychiatric phenotypes. This study suggests that many psychiatric disorders are likely to be continuous phenotypes rather than the categorical entities currently defined in diagnostic manuals, which has strong implications for genetic research in particular.

## Key Points

**Question:**

Are genetic risks for psychiatric disorders associated with subclinical population traits of these disorders?

**Findings:**

Phenotype data were available for 13 923 twin pairs at 9 years of age, 5165 pairs at 15 years of age, and 4273 pairs at 18 years of age, and genetic data were available for 13 412 individuals. Genetic risk factors for psychiatric disorders were associated with risk factors for subclinical traits; polygenic risk scores for psychiatric disorders were also significantly associated with subclinical traits.

**Meaning:**

The findings suggest that psychiatric disorders are associated with continuously distributed genetic risks throughout the general population.

## Teaser

This study assesses the association of the genetic risk for psychiatric disorders with population traits of these disorders in Sweden.

## Introduction

Psychiatric disorders are impairing and associated with genetic factors.^1^ Although clinical practice and most genetic studies follow a case-control conceptualization of these disorders, subclinical traits of these disorders are common among unaffected individuals and are as heritable as the disorders themselves based on twin methods.^2^ One common theory is that the genetic risks for psychiatric disorders are associated with these milder traits and that psychiatric disorders arise after particularly strong exposure to the same genetic risks associated with these traits.^2^

Preliminary support for this theory comes from twin and molecular genetic studies. Twin studies indicate consistent heritability of traits at varying levels of severity^3^ for autism spectrum disorder (ASD), attention-deficit/hyperactivity disorder (ADHD), and learning difficulties.^4^ A more recent UK-based twin study^5^ that used a contemporary analytic approach reported a genetic correlation of 0.70 between ASD and autistic traits. To our knowledge, this approach has not been applied to other psychiatric phenotypes, meaning that the degree of genetic correlation between most psychiatric disorders and related traits has been largely understudied using twin methods. Few twin studies have focused on the links between anxiety and major depressive disorder (MDD) with the traits of these disorders despite these being 2 of the most common psychiatric diagnoses.

Molecular genetic methods also allow estimation of genetic correlations between psychiatric disorders and subclinical traits of these disorders based on additive, common genome-wide variants. Such studies report strong genetic correlations across disorders and traits for ADHD and MDD, with a moderate estimate for ASD.^4^ This approach requires large genome-wide data sets, however, which are lacking for psychiatric traits beyond symptoms of ADHD, MDD, and ASD.^6,7^ An alternative approach is to calculate polygenic risk scores (PRSs) based on discovery genome-wide association studies (GWASs) of psychiatric disorders; PRSs capture an individual’s common variant risk for a phenotype.^8^ A review^4^ of preliminary studies reported that psychiatric disorder PRSs are associated with corresponding population traits of ASD, ADHD, obsessive-compulsive disorder (OCD), and MDD, with null or mixed findings for schizophrenia and bipolar disorder (BD).

Although some preliminary evidence supports shared genetic risks across disorders and traits for some psychiatric phenotypes, evidence is weak, mixed, or entirely lacking for many phenotypes. We aimed to assess the degree of shared genetic risks between disorders and traits for multiple psychiatric disorders. Leveraging data from a unique twin sample, with comprehensive clinical diagnostic, trait measurement, and genetic data available, enabled us to perform twin modeling and molecular genetic methods in the same sample. We used a novel twin method^5^ to estimate the genetic correlation between psychiatric diagnoses and continuous traits of these disorders. We then calculated PRSs based on recent, large-scale GWASs and tested their associations with continuous variation in related phenotypes.

## Methods

### Participants

Families of all twins born in Sweden beginning in 1992 were contacted in connection with the twins’ ninth birthday (earlier cohorts included individuals aged 12 years) and invited to participate in the Child and Adolescent Twin Study in Sweden (CATSS).^9^ The response rate was 75%. Follow-ups were conducted when the twins were 15 years of age (response rate, 61%) and 18 years of age (response rate, 59%). Exclusion criteria were brain injuries (n = 207 pairs), chromosomal syndromes (n = 35 pairs), death (n = 29 pairs), and migration (n = 100 pairs). Phenotypic data were available for 13 923 pairs at 9 years of age (1983 monozygotic male [MZM], 2641 dizygotic male [DZM], 2108 monozygotic female [MZF], 2304 dizygotic female [DZF], and 4887 dizygotic opposite-sex [DZOS]), 5165 pairs at 15 years of age (649 MZM, 854 DZM, 831 MZF, 924 DZF, and 1907 DZOS), and 4273 pairs at 18 years of age (553 MZM, 693 DZM, 722 MZF, 747 DZF, and 1558 DZOS). Zygosity was ascertained using a panel of 48 single-nucleotide polymorphisms (SNPs) or 5 questions concerning twin similarity. The latter method was only used in cases with a 95% probability of correct classification. Zygosity was reconfirmed for pairs with genotype data. All families provided written informed consent before participation, and all data were deidentified. This study received ethical approval from the Karolinska Institutet Ethical Review Board.

DNA samples (from saliva) were obtained from the CATSS participants at study enrollment. A total of 11 551 individuals with available DNA were genotyped using the Illumina PsychChip. Standard quality control and imputation procedures were performed in the sample; for details, see the article by Brikell et al.^10^ A total of 11 081 samples passed quality control assessment; MZ twins were then imputed, resulting in 13 576 samples and 6 981 993 imputed SNPs that passed all quality control assessments. After individual-level exclusions (described above), 13 412 children (50.2% females) were included in genetic analyses.

### Phenotypic Measures

#### Clinical Diagnoses

The CATSS is linked with the Swedish National Patient Register (NPR).^11^ The NPR contains *International Statistical Classification of Diseases and Related Health Problems, Tenth Revision (ICD-10)* codes for diagnoses from all visits to specialist inpatient and outpatient care in Sweden. Inpatient data were available for January 1, 1987, to December 31, 2014, and outpatient data for January 1, 2001, to December 31, 2013. Diagnoses of ASD, ADHD, intellectual disability (ID), tic disorders (TDs), OCD, anxiety disorders (ADs), and MDD were extracted. Diagnostic codes and the numbers of individuals with each diagnosis are given in Table 1. At the end of follow-up (December 31, 2014), the individuals in this study were between 9 and 22 years of age. Data analysis was performed from January 1, 2017, to September 30, 2017.

**Table 1.** Description of the CATSS Sample and Measures^a^

Phenotype	NPR Diagnosis			Study Measures		
	*ICD-10* Diagnostic Codes	Affected, No. (%)		Description of Measure	No. of Individuals With Data	
		Twin Analyses	Genetic Analyses		Twin Analyses	Genetic Analyses
ASD	F84	253 (0.9)	142 (1.1)	Ages of 9 and 12 y: parent-rated A-TAC ASD module (17 items)	27 780	13 396
ADHD	F90	824 (3.0)	440 (3.3)	Ages of 9 and 12 y: parent-rated A-TAC ADHD module (19 items)	27 759	13 391
ID	F70-F73	166 (0.6)	77 (0.6)	Ages of 9 and 12 y: parent-rated A-TAC learning module (3 items)	27 804	13 400
TDs	F95	103 (0.4)	43 (0.3)	Ages of 9 and 12 y: parent-rated A-TAC tics module (3 items)	27 791	13 396
OCD	F42	118 (0.4)	67 (0.5)	Ages of 9 and 12 y: parent-rated A-TAC compulsions module (2 items)	27 802	13 400
				Age of 18 y: self-rated BOCS (15 items)	5757	3982
ADs	F40-F41	474 (1.7)	251 (1.9)	Ages of 9 and 12 y: parent-rated SCARED (41 items)	15 589	6806
				Age of 15 y: parent-rated SDQ-E (5 items)	7663	5703
				Age of 15 y: self-rated SDQ-E (5 items)	8150	5917
				Age of 18 y: parent-rated ABCL *DSM-IV* anxiety subscale (6 items)	5160	3755
				Self-rated SCARED (38 items)	5801	4007
MDD	F32-F34	414 (1.5)	222 (1.7)	Ages of 9 and 12 y: parent-rated SMFQ (13 items)	15 873	6826
				Age of 15 y: parent-rated SDQ-E (5 items);	7663	5703
				Age of 15 y: self-rated SDQ-E (5 items);	8150	5917
				Age of 18 y: parent-rated ABCL *DSM-IV* depression subscale (15 items);	5215	3791
				Self-rated CES-D (11 items)	5518	3813
Mania	NA	NA	NA	Age of 18 y: parent-rated MDQ (13 items)	NA	3808
				Age of 18 y: self-rated MDQ (13 items)	NA	4128
Psychosis	NA	NA	NA	Age of 18 y: parent-rated APSS (7 items)	NA	5368
				Age of 18 y: self-rated APSS (7 items)	NA	5518

Abbreviations: ABCL, Adult Behavior Checklist^12^; ADs, anxiety disorders; ADHD, attention-deficit/hyperactivity disorder; APSS, Adolescent Psychotic-Like Symptom Screener^13^; ASD, autism spectrum disorder; A-TAC, Autism-Tics, AD/HD, and Other Comorbidities Inventory^14,15^; BOCS, Brief Obsessive-Compulsive Scale^16^; CATSS, Child and Adolescent Twin Study in Sweden; CES-D, Center for Epidemiologic Studies Depression Scale^17^; *ICD-10, International Statistical Classification of Diseases and Related Health Problems, Tenth Revision*; ID, intellectual disability; MDD, major depressive disorder; MDQ, Mood Disorder Questionnaire^18^; NA, not applicable; NPR, National Patient Register; OCD, obsessive-compulsive disorder; SCARED, Screen for Child Anxiety Related Emotional Disorders^19^; SDQ-E, Strengths and Difficulties Questionnaire^20^; SMFQ, Short Mood and Feelings Questionnaire^21^; TDs, tic disorders.

^a^ Diagnoses of bipolar disorder and schizophrenia were not included because of small numbers of participants with these diagnoses. There were too few items to divide the SDQ-E subscale into anxiety and depression separately.

#### Continuous Measures

Traits of ASD, ADHD, ID, TDs, OCD, ADs, and MDD were measured using continuous scales at 9 and 12 years of age. Internalizing problems (related to ADs and MDD) were then measured at 15 years of age. Traits of OCD, ADs, MDD, mania, and psychotic-like experiences were assessed at 18 years of age. Details of these measures and sample sizes are provided in Table 1, with additional details provided in eTable 1 in the Supplement.

### Analyses

#### Twin Analyses

We used joint categorical/continuous twin models to estimate the degree of etiologic overlap between continuous traits and categorical diagnoses. These models assume a normal distribution of continuous liability underlying psychiatric disorders, whereas questionnaires were treated continuously. The model partitions variance in each phenotype into additive genetic (A), nonadditive genetic (D), shared environmental (C), and nonshared environmental (E, which encompasses measurement error) components. The correlations among these components are then estimated between 2 phenotypes. The phenotypic correlations were decomposed into genetic and environmental factors to assess which factors explain the correlation between psychiatric diagnoses and traits. On the basis of twin correlations, we tested ACE or ADE models for each disorder-trait pairing. We included a sibling interaction term in ADE models (ADE-s) because these interactions can mimic the effects of D on the twin correlations.^22^ The principles of the twin design are described at length elsewhere.^23^

The ACE or ADE-s model was compared with a saturated model of the observed data. If the pattern of twin correlations differed between the continuous trait and the categorical diagnoses, both models were fitted and the best-fitting model was chosen on the basis of the lowest Bayesian Information Criteria value. More parsimonious models were tested by reducing each model by constraining certain components to equal zero and comparing these models to the ACE or ADE-s model using the likelihood ratio test; if the model fit did not deteriorate significantly, the reduced model was favored. Continuous scales were standardized by sex, whereas the association of sex with the thresholds were included in the models. Models were fitted in OpenMx.^24^ Opposite-sex twins were included, but the study was underpowered to test for sex differences. Obsessive-compulsive disorder was omitted from the twin analyses because of the small sample and low heritability.

#### PRS Analyses

Publicly available GWAS summary statistics for 8 psychiatric disorders (ie, ASD, ADHD, TDs, OCD, ADs, MDD, BD, and schizophrenia) and 3 continuously distributed psychiatric or cognitive traits (ie, ADHD symptoms, cognitive ability, and depressive symptoms) were used to derive PRSs in the CATSS individuals.^25,26,27,28,29,30,31,32,33,34^ eTable 2 in the Supplement lists these discovery data sets along with sample sizes. Discovery and target data were independent (details of PRS calculations are given in the eAppendix in the Supplement). In brief, PRSs were calculated in imputed CATSS data for each individual by scoring the number of effect alleles (weighted by the SNP effect size) across each discovery set of clumped SNPs in PLINK, version 1.9, for a range of *P* value thresholds used for SNP selection. The primary analyses are based on the threshold *P* < .50. The PRSs were standardized using *z*-score transformations; effect sizes can be interpreted as increase in risk of the outcome per SD increase in PRS. Principal component analysis was used to derive covariates to account for population stratification (eAppendix in the Supplement).

Analyses of PRSs were performed using generalized estimating equations using the R package drgee, with robust SEs, based on clustering related individuals to account for twins in the data. The principal components, sex, and age (for measures that were assessed at 9 or 12 years of age) were included as covariates. First, we tested for association between PRSs for each of the 8 discovery GWASs of psychiatric disorders and the corresponding continuously distributed trait(s). Second, these analyses were repeated after excluding individuals diagnosed with the relevant psychiatric disorder based on available information on *ICD-10* diagnoses to determine whether effects were driven primarily by individuals with clinically recognized problems. Third, we tested for associations between PRSs for each of the 3 discovery GWASs of continuously distributed population traits and the corresponding psychiatric diagnosis in the target sample. Fourth, all PRS analyses were repeated using PRSs derived on the basis of different *P* value selection thresholds to assess sensitivity. False discovery rate corrections were applied in R (using the fdr method in the function p.adjust) (R Foundation for Statistical Computing) to account for multiple testing.

#### Supplemental Analyses

Because there is some evidence of genetic specificity within ASD and ADHD trait domains,^35,36^ we reran all twin and PRS analyses for 3 specific *DSM-IV* ASD dimensions (social problems, language impairment, and behavioral inflexibility) and 2 *DSM-IV* ADHD dimensions (hyperactivity/impulsivity and inattention). These domains were assessed by dividing the Autism-Tics, AD/HD, and Other Comorbidities Inventory ASD and ADHD subscales based on prior work.^9^

## Results

### Twin Analyses

Phenotype data were available for 13 923 twin pairs (35.1% opposite sex and 31.7% same-sex females) at 9 years of age, 5165 pairs (36.9% opposite sex and 34.0% same-sex females) at 15 years of age, and 4273 pairs (36.5% opposite sex and 34.4% same-sex females) at 18 years of age. Genetic data were available for 13 412 individuals (50.2% females). Probandwise concordances for each diagnosis, which represent the probability of co-twins of probands also receiving a given diagnosis, are given in eTable 3 in the Supplement. The MZ probandwise concordances all exceeded the DZ estimates, indicating genetic associations with each diagnosis. Phenotypic correlations between disorders and related traits ranged from 0.22 for MDD to 0.66 for ID (mean estimate, 0.40) (Table 2). The MZ twin correlations were higher than the DZ correlations within each disorder and trait and across disorder-trait pairs, suggesting that each phenotype and the covariance between was associated with genetic factors.

**Table 2.** Phenotypic and Twin Correlations Across Disorders and Continuous Traits^a^

Disorder, Outcome (Age, y)	Correlation Coefficient (95% CI)						
	rPH	Continuous Scale		Categorical Diagnosis		Cross-Trait	
		MZ	DZ	MZ	DZ	MZ	DZ
ASD							
A-TAC ASD (9 and 12)	0.45 (0.42 to 0.48)	0.74 (0.72 to 0.75)	0.27 (0.25 to 0.28)	0.81 (0.67 to 0.90)	0.31 (0.18 to 0.43)	0.39 (0.33 to 0.45)	0.14 (0.08 to 0.21)
ADHD							
A-TAC ADHD (9 and 12)	0.52 (0.50 to 0.54)	0.69 (0.68 to 0.71)	0.23 (0.21 to 0.25)	0.88 (0.83 to 0.92)	0.44 (0.37 to 0.50)	0.47 (0.43 to 0.51)	0.17 (0.13 to 0.21)
ID							
A-TAC learning (9 and 12)	0.66 (0.63 to 0.69)	0.72 (0.70 to 0.73)	0.13 (0.11 to 0.15)	0.93 (0.84 to 0.98)	0.30 (0.15 to 0.44)	0.54 (0.45 to 0.62)	0.12 (0.03 to 0.21)
TDs							
A-TAC tics (9 and 12)	0.48 (0.44 to 0.52)	0.44 (0.41 to 0.46)	0.11 (0.09 to 0.13)	0.64 (0.39 to 0.82)	−0.26 (−0.68 to 0.28)	0.32 (0.20 to 0.43)	0.09 (−0.02 to 0.20)
ADs							
SCARED (9)	0.30 (0.24 to 0.36)	0.66 (0.64 to 0.68)	0.37 (0.35 to 0.39)	0.67 (0.55 to 0.77)	0.30 (0.18 to 0.42)	0.39 (0.22 to 0.52)	0.14 (0.04 to 0.25)
SDQ-E parent-rated (15)	0.36 (0.32 to 0.41)	0.48 (0.44 to 0.52)	0.22 (0.18 to 0.25)	0.66 (0.53 to 0.76)	0.30 (0.18 to 0.41)	0.33 (0.23 to 0.43)	0.15 (0.06 to 0.23)
SDQ-E self-rated (15)	0.26 (0.21 to 0.31)	0.44 (0.39 to 0.48)	0.18 (0.15 to 0.22)	0.66 (0.53 to 0.76)	0.30 (0.18 to 0.42)	0.23 (0.11 to 0.35)	0.07 (−0.02 to 0.16)
ABCL anxiety (18)	0.40 (0.35 to 0.45)	0.57 (0.53 to 0.61)	0.32 (0.28 to 0.36)	0.65 (0.52 to 0.76)	0.30 (0.18 to 0.41)	0.30 (0.19 to 0.40)	0.16 (0.06 to 0.25)
SCARED (18)	0.42 (0.36 to 0.47)	0.49 (0.44 to 0.54)	0.17 (0.12 to 0.22)	0.65 (0.53 to 0.76)	0.30 (0.18 to 0.41)	0.42 (0.30 to 0.52)	0.17 (0.08 to 0.27)
MDD							
SMFQ (9)	0.22 (0.13 to 0.30)	0.55 (0.52 to 0.57)	0.29 (0.27 to 0.31)	0.68 (0.56 to 0.77)	0.37 (0.24 to 0.48)	0.56 (0.33 to 0.70)	0.03 (−0.10 to 0.17)
SDQ-E parent-rated (15)	0.37 (0.32 to 0.42)	0.48 (0.44 to 0.52)	0.22 (0.18 to 0.25)	0.69 (0.57 to 0.78)	0.34 (0.21 to 0.46)	0.22 (0.12 to 0.32)	0.15 (0.06 to 0.24)
SDQ-E self-rated (15)	0.28 (0.22 to 0.33)	0.45 (0.40 to 0.49)	0.18 (0.15 to 0.22)	0.68 (0.56 to 0.77)	0.36 (0.23 to 0.48)	0.30 (0.18 to 0.41)	0.09 (−0.02 to 0.19)
ABCL depression (18)	0.44 (0.40 to 0.49)	0.52 (0.48 to 0.56)	0.26 (0.21 to 0.30)	0.66 (0.54 to 0.77)	0.35 (0.22 to 0.47)	0.34 (0.25 to 0.43)	0.14 (0.04 to 0.23)
CES-D (18)	0.44 (0.39 to 0.49)	0.47 (0.41 to 0.51)	0.19 (0.14 to 0.23)	0.68 (0.56 to 0.78)	0.36 (0.23 to 0.47)	0.32 (0.20 to 0.44)	0.17 (0.07 to 0.26)

Abbreviations: ABCL, Adult Behavior Checklist; ADs, anxiety disorders; ASD, autism spectrum disorder; ADHD, attention-deficit/hyperactivity disorder; A-TAC, Autism-Tics, AD/HD, and Other Comorbidities Inventory; CES-D, Center for Epidemiologic Studies Depression Scale; DZ, dizygotic; ID, intellectual disability; MDD, major depressive disorder; MZ, monozygotic; rPH, phenotypic correlation between continuous and categorical diagnosis; SCARED, Screen for Child Anxiety Related Emotional Disorders; SDQ-E, Strengths and Difficulties Questionnaire, emotional problems subscale; SMFQ, Short Mood and Feelings Questionnaire; TDs, tic disorders.

^a^ The table gives the cross-twin correlations for the continuous scale and categorical diagnosis followed by the cross-trait cross-twin correlations, with ranges in parentheses. All correlations given in the table were estimated from a saturated model with constraints on the means and variances for continuous traits and on the thresholds for categorical diagnoses.

For all traits, AE-s or AE models fit best except for ADs at 9 and 12 years of age, where there was a small, significant C estimate (eTable 4 in the Supplement). Variance components and etiologic correlations from each model are given in eTable 5 in the Supplement. All measures and diagnoses except for the Strengths and Difficulties Questionnaire at 15 years of age and self-reported MDD at 18 years of age were under strong genetic influence. The genetic and nonshared environmental correlations are shown in the Figure. Point estimates of genetic correlation varied: 0.48 for ASD (95% CI, 0.44-0.53), 0.56 for ADHD (95% CI, 0.53-0.59), 0.69 for ID (95% CI, 0.64-0.73), 0.61 for TDs (95% CI, 0.51-0.80), 0.46 to 0.57 for ADs (95% CI, 0.34-0.58 and 0.46-0.68), and 0.33 to 0.58 for MDD (95% CI, 0.19-0.47 and 0.52-0.63). For anxiety and MDD, higher genetic correlations were estimated at 18 years of age. However, most of the CIs around the genetic correlations overlapped. Shared genetic factors explained 60% to 100% (mean estimate, 80.7%) of the phenotypic covariance between each trait and diagnosis (eTable 5 in the Supplement).

**Figure.**
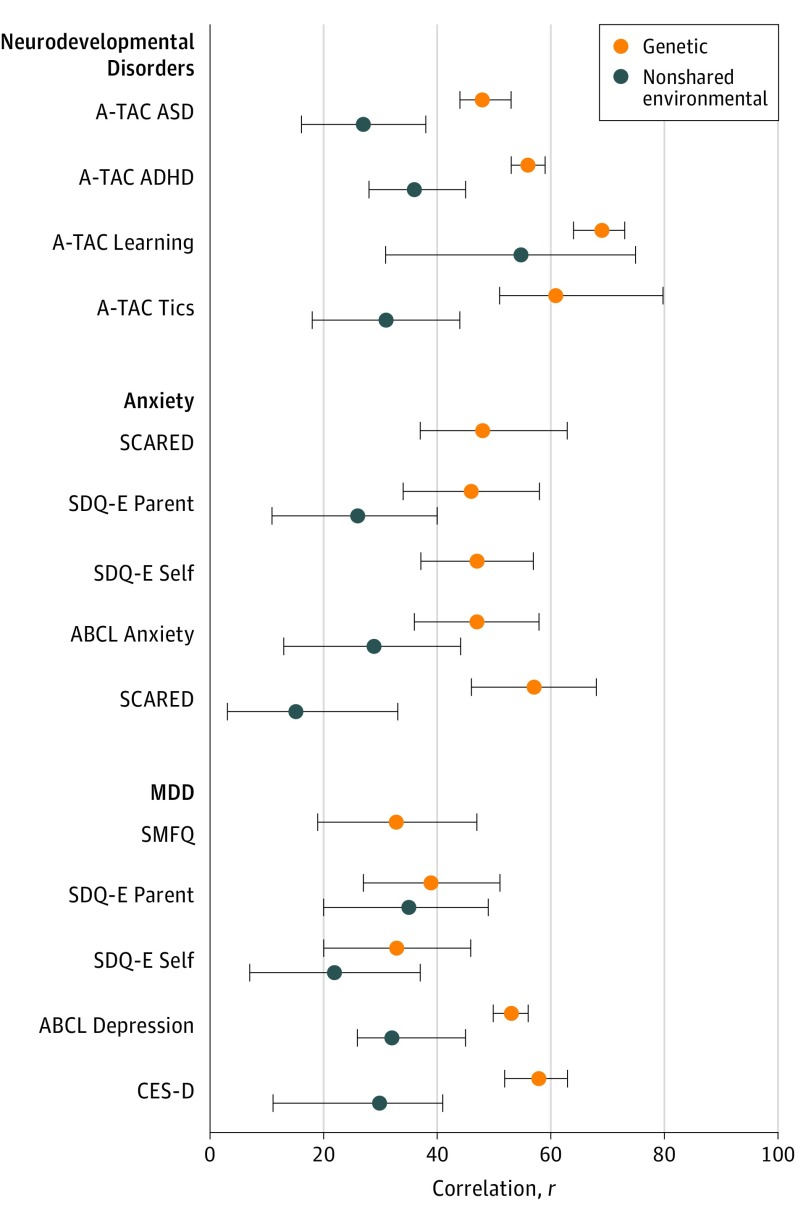
Genetic and Nonshared Environmental Correlations Between Psychiatric Diagnoses and Traits From the Best-Fitting Twin Models ABCL, Adult Behavior Checklist; ADHD, attention-deficit/hyperactivity disorder; ASD, autism spectrum disorder; A-TAC: Autism-Tics, AD/HD, and Other Comorbidities Inventory; CES-D, Center for Epidemiologic Studies Depression Scale; MDD, major depressive disorder; SCARED, Screen for Child Anxiety Related Emotional Disorders; SDQ-E, Strengths and Difficulties Questionnaire, emotional problems subscale; SMFQ, Short Mood and Feelings Questionnaire.

Analyses of specific ASD and ADHD domains are given in eTables 6 and 7 in the Supplement. All ASD trait domains displayed moderate phenotypic (mean estimate, 0.41; range, 0.43-0.48) and genetic correlations (mean estimate, 0.46; range, 0.43-0.47) with ASD, whereas both ADHD dimensions displayed moderate phenotypic (mean estimate, 0.49; range, 0.45-0.53) and genetic (mean estimate, 0.53; range, 0.49-0.57) correlations with ADHD.

### PRS Analyses

The PRSs for psychiatric disorders were associated with related traits for all disorders except BD (Table 3). At 9 years of age, ASD PRSs were associated with autistic traits (β [SE] = 0.04 [0.01]), ADHD PRSs were associated with ADHD traits (β [SE] = 0.27 [0.03]), and TD PRSs were associated with tic problems (β [SE] = 0.02 [0.004]). The OCD PRSs were associated with obsessive-compulsive symptoms at 18 years of age (β [SE] = 0.13 [0.05]) but not at 9 and 12 years of age (β [SE] = 0.002 [0.002]). The AD PRSs were associated with anxiety traits at 9 years of age (β [SE] = 0.18 [0.08]), parent-rated internalizing traits at 15 years of age (β [SE] = 0.07 [0.02]), and self-rated traits at 18 years of age (β [SE] = 0.40 [0.17]) but not with self-rated internalizing traits at 15 years of age (β [SE] = 0.06 [0.03]) and parent-rated symptoms at 18 years of age (β [SE] = 0.04 [0.03]). The MDD PRSs were associated with all measures of depressive symptoms (β [SE] = 0.10 [0.03] at 9 years of age, β [SE] = 0.11 [0.02] at 15 years of age parent-rated, β [SE] = 0.11 [0.03] at 15 years of age self-rated, β [SE] = 0.25 [0.06] at 18 years of age parent-rated; β [SE] = 0.41 [0.10] at 18 years of age self-rated). Schizophrenia PRSs were associated with psychotic traits at 18 years of age (β [SE] = 0.02 [0.01]).

**Table 3.** Association of PRSs With Related Continuous Outcomes

Discovery PRS, Outcome (Age, y)	Full Sample			Excluding Those With *ICD-10* Diagnoses			
	β (SE)	*P* Value	*R*^2^	β (SE)	*P* Value		*R*^2^
ASD							
A-TAC ASD (9 and 12)	0.043 (0.014)	5.4 × 10^−3^^a^	9.5 × 10^−4^	0.036 (0.013)	6.7 × 10^−3^^a^		8.2 × 10^−4^
ADHD							
A-TAC ADHD (9 and 12)	0.268 (0.029)	5.9 × 10^−19^^b^	8.4 × 10^−3^	0.205 (0.027)	2.2 × 10^−13^^b^		6.2 × 10^−3^
TDs							
A-TAC tics (9 and 12)	0.015 (0.004)	6.6 × 10^−4^^b^	1.2 × 10^−3^	0.016 (0.004)	5.3 × 10^−4^^b^		1.3 × 10^−3^
OCD							
A-TAC OC traits (9 and 12)	0.002 (0.002)	0.333	1.2 × 10^−4^	0.002 (0.002)	0.262		1.4 × 10^−4^
BOCS (18)	0.126 (0.047)	0.014^c^	2.3 × 10^−3^	0.132 (0.046)	6.7 × 10^−3^^a^		2.6 × 10^−3^
ADs							
SCARED (9)	0.180 (0.078)	0.033^c^	9.1 × 10^−4^	0.186 (0.078)	0.023^c^		1.0 × 10^−3^
SDQ-E parent-rated (15)	0.069 (0.023)	5.4 × 10^−3^^a^	1.9 × 10^−3^	0.054 (0.022)	0.018^c^		1.3 × 10^−3^
SDQ-E self-rated (15)	0.061 (0.030)	0.060	7.3 × 10^−4^	0.055 (0.030)	0.077		6.1 × 10^−4^
ABCL anxiety (18)	0.043 (0.030)	0.186	6.6 × 10^−4^	0.032 (0.029)	0.262		4.2 × 10^−4^
SCARED (18)	0.404 (0.171)	0.031^c^	1.5 × 10^−3^	0.344 (0.166)	0.047^c^		1.2 × 10^−3^
MDD							
SMFQ (9)	0.095 (0.028)	2.1 × 10^−3^^a^	2.0 × 10^−3^	0.095 (0.028)	1.7 × 10^−3^^a^		2.0 × 10^−3^
SDQ-E parent-rated (15)	0.105 (0.023)	5.3 × 10^−5^^b^	4.4 × 10^−3^	0.079 (0.022)	1.3 × 10^−3^^a^		2.7 × 10^−3^
SDQ-E self-rated (15)	0.105 (0.030)	1.3 × 10^−3^^a^	2.1 × 10^−3^	0.087 (0.029)	6.5 × 10^−3^^a^		1.5 × 10^−3^
ABCL depression (18)	0.249 (0.055)	5.3 × 10^−5^^b^	7.3 × 10^−3^	0.186 (0.049)	6.3 × 10^−4^^b^		5.1 × 10^−3^
CES-D (18)	0.408 (0.100)	2.3 × 10^−4^^b^	4.8 × 10^−3^	0.343 (0.097)	1.3 × 10^−3^^a^		3.6 × 10^−3^
BD							
MDQ parent-rated (18)	-0.045 (0.061)	0.478	2.3 × 10^−4^	NA^d^	NA^d^	NA^d^	
MDQ self-rated (18)	-0.054 (0.058)	0.388	2.4 × 10^−4^	NA^d^	NA^d^	NA^d^	
Schizophrenia							
APSS parent-rated (18)	0.024 (0.010)	0.031^c^	1.5 × 10^−3^	NA^d^	NA^d^	NA^d^	
APSS self-rated (18)	0.062 (0.021)	8.4 × 10^−3^^a^	1.7 × 10^−3^	NA^d^	NA^d^	NA^d^	

Abbreviations: ABCL, Adult Behavior Checklist; ADs, anxiety disorders; ADHD, attention-deficit/hyperactivity disorder; APSS, Adolescent Psychotic-Like Symptom Screener; ASD, autism spectrum disorder; A-TAC, Autism-Tics, AD/HD, and Other Comorbidities Inventory; BD, bipolar disorder; BOCS, Brief Obsessive-Compulsive Scale; CES-D, Center for Epidemiologic Studies Depression Scale; *ICD-10, International Statistical Classification of Diseases and Related Health Problems, Tenth Revision*; MDD, major depressive disorder; MDQ, Mood Disorder Questionnaire; NA, not applicable; OCD, obsessive-compulsive disorder; PRS, polygenic risk score; SCARED, Screen for Child Anxiety Related Emotional Disorders; SDQ-E, Strengths and Difficulties Questionnaire, emotional problems subscale; SMFQ, Short Mood and Feelings Questionnaire; TDs, tic disorders.

^a^ False discovery rate *P* < .01.

^b^ False discovery rate *P* < .001.

^c^ False discovery rate *P* < .05.

^d^ Sample too young to exclude individuals diagnosed with BD or schizophrenia. The PRSs were derived using common variants with *P* < .50 in the discovery data.

After removing individuals diagnosed with the relevant psychiatric disorder, the results remained significant for ASD, ADHD, TDs, OCD, ADs, and MDD, although the effect sizes decreased (Table 3). All estimates of variance explained were modest (mean, 0.23%; range, 0.01%-0.84%).

In the analysis of PRSs for quantitative traits associated with psychiatric diagnoses (Table 4), PRSs for depressive symptoms were associated with MDD diagnosis (odds ratio [OR], 1.16; 95% CI, 1.02-1.32); PRSs for traits of ADHD (OR, 1.09; 95% CI, 0.98-1.22) and general cognitive ability (OR, 0.97; 95% CI, 0.76-1.23) were not associated with related diagnoses (ADHD and ID, respectively).

**Table 4.** Association of Continuous Trait PRSs With Related Binary Outcomes

Discovery PRS	Outcome (Diagnosis)	No. of Individuals	OR (95% CI)	*P* Value	*R*^2^
ADHD traits	*ICD-10* (ADHD)	13 412	1.09 (0.98-1.22)	.13	9.9 × 10^−4^
General cognition	*ICD-10* (ID)	13 412	0.97 (0.76-1.23)	.78	9.6 × 10^−5^
Depressive traits	*ICD-10* (MDD)	13 412	1.16 (1.02-1.32)	.04^a^	2.3 × 10^−3^

Abbreviations: ADHD, attention-deficit/hyperactivity disorder; *ICD-10, International Statistical Classification of Diseases and Related Health Problems, Tenth Revision*; ID, intellectual disability; MDD, major depressive disorder; OR, odds ratio; PRS, polygenic risk score.

^a^ False discovery rate *P* < .05.

Secondary analyses for ASD and ADHD PRSs associated with specific trait domains are given in eTable 8 in the Supplement. The analyses of these subdomains were consistent with the analyses of total ASD (social: β [SE] = 0.011 [0.006], language: β [SE] = 0.011 [0.006], flexibility: β [SE] = 0.022 [0.005]) and ADHD (hyperactivity/impulsivity: β [SE] = 0.14 [0.015], inattention: β [SE] = 0.130 [0.016]) trait scores except that, for ASD, only the estimate for flexibility remained significant after excluding individuals with ASD diagnoses.

All analyses were repeated using PRSs derived on the basis of different *P* value thresholds for SNP inclusion (eFigure in the Supplement). The pattern of results was consistent in these sensitivity analyses, but 1 result (ie, ADs at 18 years of age) was not statistically significant after false discovery rate correction.

## Discussion

Using a unique, large, genotyped twin sample, we tested for genetic associations between clinical psychiatric diagnoses and related traits. All disorders analyzed using novel twin models (ASD, ADHD, TDs, ID, ADs, and MDD) showed modest to strong genetic correlations with related traits. Squaring these correlations gives the proportion of genetic variance shared between 2 traits; thus, our findings suggest that at least a modest proportion of genetic factors associated with clinical psychiatric disorders are associated with continuous variation in milder traits of these disorders. This finding replicated the results of an earlier study^5^ of ASD and extended the method to many other disorders. Common variant PRS analyses supported these results, revealing an association of shared risks between ASD, ADHD, TDs, OCD, ADs, MDD, and schizophrenia and related traits, even after excluding individuals who had received a diagnosis, where possible. These converging results using 2 contemporary methods revealed that many psychiatric disorders may share genetic risks with continuous symptom dimensions in the population.

Our study went beyond traditional twin studies by directly estimating the genetic correlation between psychiatric disorders and continuous traits; we also assessed the association between disorder PRSs and continuous traits in the same sample. Dichotomous definitions of psychiatric disorders may not be optimal for all studies of these phenotypes. Our results indicate that moving beyond dichotomous definitions of psychiatric disorders to joint analyses of disorders and traits may increase statistical power and yield insights into the biology of these traits. The value of such an approach was demonstrated by a recent ADHD GWAS.^26^ Studies of traits in community-based samples may also be more representative than clinical samples while generating results that, to a degree, generalize to clinical populations.

However, genetic correlations in the twin analyses were less than 1. Associations between PRSs and traits had small effect sizes (which is typical of PRS studies). Thus, only a proportion of genetic risks were shared across disorders and traits. Although twin methods capture all sources of inherited genetic risk, PRSs are limited to additive common effects. Rare genetic variants may have a more deleterious effect than common variants; however, several studies have demonstrated genetic overlap from rare variants across disorders and continuous measures of ASD^37^ and ID.^38^ Correlations between environmental factors associated with psychiatric disorders and traits were also lower than the genetic correlations, suggesting that environmental factors associated with psychiatric disorders may be more unique to psychiatric disorders than genetic factors. Future work identifying risk factors that are not shared between psychiatric traits and disorders may thus help elucidate why some individuals present with mild traits, whereas others manifest clinically severe problems.

### Strengths and Limitations

A unique strength of our study was that we were able to perform both twin and molecular genetic analyses in 1 cohort. Linkage with nationwide patient records enabled us to focus on clinical diagnoses of psychiatric disorders as opposed to percentile-based cutoffs or screening diagnoses. This study design allowed the exclusion of individuals diagnosed with psychiatric disorders from PRS analyses, thus ruling out the possibility that observed effects were driven by individuals with clinically recognized problems. Assessments from multiple ages and raters led to a wealth of information on psychiatric phenotypes.

Nonetheless, the sample was young. Although many disorders develop during childhood and adolescence, disorders such as schizophrenia, BD, and severe depression become more common with age. Because few of the participants had passed through the periods of high risk for these disorders, we could not perform twin analyses of schizophrenia or BD or exclude those individuals from PRS analyses. Studies of older individuals are thus needed as a next step. In addition, the NPR covers specialist care; thus, diagnoses ascribed through primary care were likely missed.

Specific limitations of the genetic analyses include modest sample sizes and associated low power of several of the discovery GWAS analyses used to derive PRSs; for certain phenotypes, these limitations may have led to less robust results (eg, for ADs and BD). Nonrandom attrition, previously reported to affect genetic studies,^39^ may have decreased the observed effect sizes, particularly at later ages. In addition, the estimates of variance explained were low although typical of PRS analyses (eg, PRSs explain only 5.5% of the variance in ADHD clinical case status^26^) because PRSs capture only the most strongly associated common variants and rely on discovery GWAS power to accurately estimate SNP effects. Although the PRS results showed the presence of associations between genetic risk for disorders and traits consistent with the twin analyses, the degree to which common genetic risks are shared is unclear from our study. Future studies that use larger GWAS data sets and other methods are needed to estimate genetic correlations from molecular genetic data.

We did not have the statistical power to divide the cases by severity or diagnostic subtype. Thus, we cannot conclude that all levels of disorder severity share genetic risks with milder traits. This topic will be an important focus in future research because there is some evidence that severe ID is genetically independent from cognitive abilities and milder ID.^40^ In addition, all the continuous measures used in this study were designed to assess potentially problematic behaviors. As such, we cannot extrapolate our results to the very low positive end of each trait distribution. Studies that use measures that are sensitive to lower scores are needed.^41^

## Conclusions

Although our results do not rule out the possibility that some genetic factors are not shared between psychiatric disorders and milder traits of these disorders, they suggest that a proportion of genetic risks associated with psychiatric disorders are also associated with milder traits of these disorders. Future studies are needed to replicate our findings in older individuals and to test whether more severe forms of psychiatric disorders also share genetic risks with milder traits.

## Notes

Supplement. eAppendix. Supplemental Methods eTable 1. Additional Description of the Study Measures eTable 2. Summary of Discovery Data Sets Used to Derive Polygenic Risk Scores eTable 3. Probandwise Concordances eTable 4. Twin Model Fit Statistics for Different Models Comparing the Associations Between Clinical Diagnosis and Continuous Traits eTable 5. Parameter Estimates From the Best-Fitting Twin Models of the Associations Between Continuous Traits and Clinical Diagnoses eTable 6. Twin Fit Statistics for Diagnostic Subdomains eTable 7. Twin Model Estimates for Diagnostic Subdomains eTable 8. Association of Polygenic Risk Scores for ADHD and ASD With Traits of Diagnostic Subdomains eFigure. Results of Sensitivity Analyses
