# Supplementary material for: Association of Genetic Risk Factors for Psychiatric Disorders and Traits of These Disorders in a Swedish Population Twin Sample
Source: JAMA Psychiatry. 2018 Dec 19;76(3):280–9. doi: 10.1001/jamapsychiatry.2018.3652 (PMC6439816; doi:10.1001/jamapsychiatry.2018.3652)
Supplement: Supplement. — eAppendix. Supplemental Methods eTable 1. Additional Description of the Study Measures eTable 2. Summary of Discovery Data Sets Used to Derive Polygenic Risk Scores eTable 3. Probandwise Concordances eTable 4. Twin Model Fit Statistics for Different Models Comparing the Associations Between Clinical Diagnosis and Continuous Traits eTable 5. Parameter Estimates From the Best-Fitting Twin Models of the Associations Between Continuous Traits and Clinical Diagnoses eTable 6. Twin Fit Statistics for Diagnostic Subdomains eTable 7. Twin Model Estimates for Diagnostic Subdomains eTable 8. Association of Polygenic Risk Scores for ADHD and ASD With Traits of Diagnostic Subdomains eFigure. Results of Sensitivity Analyses [file jamapsychiatry-76-280-s001.pdf]

Supplementary Online Content

Taylor MJ, Martin J, Lu Y, et al. Association of Genetic Risk Factors for Psychiatric Disorders and Traits of These Disorders in a Swedish Population Twin Sample. *JAMA Psychiatry.* Published online December 19, 2018. doi:10.1001/jamapsychiatry.2018.3652

eAppendix. Supplemental Methods

eTable 1. Additional Description of the Study Measures

eTable 2. Summary of Discovery Data Sets Used to Derive Polygenic Risk Scores

eTable 3. Probandwise Concordances

eTable 4. Twin Model Fit Statistics for Different Models Comparing the Associations Between Clinical Diagnosis and Continuous Traits

eTable 5. Parameter Estimates From the Best-Fitting Twin Models of the Associations Between Continuous Traits and Clinical Diagnoses

eTable 6. Twin Fit Statistics for Diagnostic Subdomains

eTable 7. Twin Model Estimates for Diagnostic Subdomains

eTable 8. Association of Polygenic Risk Scores for ADHD and ASD With Traits of Diagnostic Subdomains

eFigure. Results of Sensitivity Analyses

This supplementary material has been provided by the authors to give readers additional information about their work.

© 2018 American Medical Association. All rights reserved.

eAppendix. Supplemental Methods

Polygenic risk score calculation

Publicly available genome-wide association study (GWAS) summary statistics for 8 psychiatric disorders and 3 continuously distributed psychiatric or cognitive traits[1–10] were used to derive polygenic risk scores (PRS) in the CATSS individuals. Where multiple discovery GWAS summary statistics files on different ancestry groups were available, the GWAS results restricted to individuals of European ancestry were used. The discovery and target data were independent. CATSS data have been genotyped recently[11] and have not been previously contributed to any GWAS consortia. The cohort is too young to have previously taken part in published research studies of adult psychiatric disorders. Additionally, care was taken to ensure that the discovery GWAS did not include any individuals who may be related to those in the target CATSS data by excluding Swedish data (e.g. the PAGES data from the autism GWAS). Each discovery GWAS results file was filtered based on available information for minor allele frequency (MAF>0.05) and imputation quality (INFO>0.8). Indels, asymmetric/ambiguous (AT, TA, CG, GC), multi-allelic and duplicate position SNPs were excluded. Where necessary, SNPs were lifted over to genome build hg19 using the UCSC Liftover tool. SNPs were matched across the target and discovery data based on chromosomal position.

Next, LD-clumping was run using the 1000-Genomes reference population (which was used for imputation of the CATSS data), to obtain a relatively independent set of autosomal SNPs for each set of discovery results, while retaining the most significant SNP in each LD block and maximizing the overlap of SNPs across the discovery and target data. The following parameters were applied in PLINK.v.1.9: --clump-kb 1000 --clump-r2 0.1. The number of clumped SNPs for each discovery study varied from 55,072-89,358.

PRS were then calculated for each individual in CATSS by scoring the number of effect alleles (weighted by the SNP log of the odds ratio or SNP beta) across each discovery set of clumped SNPs in PLINK.v.1.9 (using the command --score no-mean-imputation). The p-value thresholds used for SNP selection were p<1, p<0.5, p<0.1, p<0.05, p<0.01, and p<0.001. The primary analyses are based on the threshold p<0.5. Scores were derived in imputed best guess genotype data after filtering out SNPs with MAF<0.05 and INFO<0.8. The PRS were standardized using z-score transformations; effect sizes can be interpreted as increase in risk of the outcome, per standard deviation increase in PRS. Nagelkerke R^2 differences between null and full models were calculated to obtain estimates of variance explained.

Principal components analysis

A principal components analysis (PCA) was performed to derive population covariates (PCs) in all individuals. First, common autosomal genotyped SNPs passing all QC (N=287,655) were LD-pruned after removing SNPs located in long-range LD regions. Next, relatives were identified using an identity-by-descent analysis

© 2018 American Medical Association. All rights reserved.

and one of each pair of related individuals (pi-hat>0.2) were temporarily excluded. Allele frequencies were obtained for the set of LD-pruned SNPs for unrelated individuals (N=83,648 SNPs and N=7,112 individuals). PCs were then estimated by calculating variant weights on unrelated individuals and then projecting remaining samples to the PC scales set by these unrelated individuals. All analyses were carried out using PLINK.v.1.9. The first 10 principal components were used as covariates for subsequent analyses.

References

1. Grove, J. *et al.* Common risk variants identified in autism spectrum disorder. *bioRxiv* (2017).
2. Demontis, D. *et al.* Discovery Of The First Genome-Wide Significant Risk Loci For ADHD. *bioRxiv* (2017). doi:doi: https://doi.org/10.1101/145581
3. Arnold, P. D. *et al.* Revealing the complex genetic architecture of obsessive–compulsive disorder using meta-analysis. *Mol. Psychiatry* (2017). doi:10.1038/mp.2017.154
4. Otowa, T. *et al.* Meta-analysis of genome-wide association studies of anxiety disorders. *Mol. Psychiatry* **21,** 1391–1399 (2016).
5. Wray, N. R. *et al.* Genome-wide association analyses identify 44 risk variants and refine the genetic architecture of major depression. *Nat. Genet.* (2018). doi:10.1038/s41588-018-0090-3
6. Sklar, P. *et al.* Large-scale genome-wide association analysis of bipolar disorder identifies a new susceptibility locus near ODZ4. *Nat. Genet.* **43,** 977–983 (2011).
7. Schizophrenia Working Group of the Psychiatric Genomics Consortium. Biological insights from 108 schizophrenia-associated genetic loci. *Nature* **511,** 421–427 (2014).
8. Middeldorp, C. M. *et al.* A Genome-Wide Association Meta-Analysis of Attention-Deficit/Hyperactivity Disorder Symptoms in Population-Based Paediatric Cohorts. *J. Am. Acad. Child Adolesc. Psychiatry* **55,** 896–905 (2016).
9. Okbay, A. *et al.* Genetic variants associated with subjective well-being, depressive symptoms, and neuroticism identified through genome-wide analyses. *Nat. Genet.* **48,** 624–633 (2016).
10. Trampush, J. W. *et al.* GWAS meta-analysis reveals novel loci and genetic correlates for general cognitive function: a report from the COGENT consortium. *Mol. Psychiatry* **22,** 336–345 (2017).
11. Brikell, I. *et al.* The contribution of common genetic risk variants for ADHD to a general factor of childhood psychopathology. *Mol. Psychiatry* (2018). doi:10.1038/s41380-018-0109-2
12. Achenbach, T. M., Dumenci, L. & Rescorla, L. A. Ratings of relations between DSM-IV diagnostic categories and items of the Adult Self-Report (ASR) and Adult Behavior Checklist (ABCL). *Res. Cent.*

© 2018 American Medical Association. All rights reserved.

Child. Youth&Families (2003).

13. Hansson, S. L. *et al.* Psychiatric telephone interview with parents for screening of childhood autism – tics, attention-deficit hyperactivity disorder and other comorbidities (A–TAC). *Br. J. Psychiatry* **187,** (2005).

14. Larson, T. *et al.* The autism--tics, AD/HD and other comorbidities inventory (A-TAC): further validation of a telephone interview for epidemiological research. *BMC Psychiatry* **10,** 1 (2010).

15. Laurens, K. *et al.* Community screening for psychotic-like experiences and other putative antecedents of schizophrenia in children aged 9–12 years. *Schizophr. Res.* **90,** 130–146 (2007).

16. Bejerot, S. *et al.* The Brief Obsessive-Compulsive Scale (BOCS): a self-report scale for OCD and obsessive-compulsive related disorders. *Nord. J. Psychiatry* **68,** 549–59 (2014).

17. Radloff, L. S. The CES-D Scale. *Appl. Psychol. Meas.* **1,** 385–401 (1977).

18. Wagner, K. D. *et al.* Validation of the Mood Disorder Questionnaire for bipolar disorders in adolescents. *J. Clin. Psychiatry* **67,** 827–30 (2006).

19. Hale, W. W., Raaijmakers, Q., Muris, P. & Meeus, W. Psychometric Properties of the Screen for Child Anxiety Related Emotional Disorders (SCARED) in the General Adolescent Population. *J. Am. Acad. Child Adolesc. Psychiatry* (2005). doi:10.1097/00004583-200503000-00013

20. Goodman, R. The Strengths and Difficulties Questionnaire: a research note. *J. Child Psychol. Psychiatry.* **38,** 581–6 (1997).

21. Angold, A. *et al.* The development of a short questionnaire for use in epidemiological studies of depression in children and adolescents. *International Journal of Methods in Psychiatric Research* (1995). doi:1049-8931/95/040251-12

© 2018 American Medical Association. All rights reserved.

eTable 1. Additional Description of the Study Measures

Phenotype	Measure	Description	Range of scale
**ASD**	A-TAC ASD module (17 items)	Social interaction (6), communication (6) & flexible behavior (5) items	0-17
**ADHD**	A-TAC ADHD module (19 items)	Inattentive (9) & hyperactive-impulsive (10) items	0-19
**ID**	A-TAC Learning module (3 items)	Reading difficulties (1), maths difficulties (1) & general learning delay (1) items	0-3
**TD**	A-TAC Tics module (3 items)	Motor (1) & vocal (2) tic items	0-3
**OCD**	ATAC Compulsions module (2 items); BOCS (15 items)	A-TAC: Compulsion (1) & obsession (1) items; BOCS: Contamination (2), checking (1), superstition (2), morality (1), harming obsessions (2), sexual obsessions (1), somatic obsessions (1), symmetry/ordering (2), rituals (1), hoarding (1), self-harm (1)	0-2; 0-15
**ANX**	SCARED (41 items); SDQ Emotion subscale (5 items); ABCL DSM-IV Anxiety subscale (6 items); SCARED (38 items)	SCARED: Panic (13), general anxiety (9), separation anxiety (8), school phobia (4) & social phobia (7) items; Panic (13), general anxiety (9), separation anxiety (8), school phobia (4) & social phobia (4) items (social phobia has 7 items in the 41 item SCARED); SDQ: somatic symptoms (1), worries (1), unhappiness (1), nervousness (1), fearfulness (2); ABCL: worries (3), fears (1), nervousness (1) items	0-82; 0-10; 0-12; 0-76
**MDD**	SMFQ (13 items); SDQ Emotion subscale (5 items); ABCL DSM-IV Depression subscale (15 items); CES-D (11 items)	SMFQ: low mood (1), anhedonia (1), tired (1), restless (1), low self worth (6), crying (1), cognitive difficulties (1), loneliness (1) items; SDQ: see ANX; ABCL: crying (1), self-harm/suicide (2), low appetite (1), negative self-worth (2), guilt (1),disrupted sleep (3), anhedonia (1), difficulties making decisions (1), passive (1), low energy (1), low mood (1) items; CESD: appetite (1), low mood (3), tiredness (1), disturbed sleep (1), loneliness (1), others unfriendly (2), enjoyment (1), behavior inactivation (1) items	0-26; 0-10; 0-30; 0-33
**Mania**	MDQ (13 items)	Hyperactivity (2), irritability (1), self confidence (1), reduced need for sleep (1), talkativeness (1), racing thoughts (1), distracted (1), high energy (1), hyper-sociability (1), hypersexual (1), atypical behavior (1), spending too much money (1)	0-13
**Psychosis**	APSS (7 items)	Paranoia (4), grandiosity (2), visual hallucinations (1)	0-7

© 2018 American Medical Association. All rights reserved.

ASD: autism spectrum disorders; ADHD: attention-deficit/hyperactivity disorder; ID: intellectual disability; TD: tic disorders; OCD: obsessive-compulsive disorder; ANX: anxiety disorders; MDD: major depressive disorder; ABCL: Adult Behavior Checklist[12]; A-TAC: Autism-Tics, AD/HD, and other Comorbidities inventory[13,14]; APSS: Adolescent Psychotic-Like Symptoms Screener[15]; BOCS: Brief Obsessions and Compulsions Scale[16]; CES-D: Center for Epidemiologic Studies Depression scale[17]; MDQ: Mood Disorders Questionnaire[18]; NPR: National Patient Register; SCARED: Screen for Anxiety Related Disorders[19]; SDQ: Strengths and Difficulties Questionnaire[20]; SMFQ: Short Moods and Feelings Questionnaire[21].

© 2018 American Medical Association. All rights reserved.

eTable 2: Summary of Discovery Data Sets Used to Derive Polygenic Risk Scores

Phenotype	Consortium	Sample size	Publication
**ASD**	PGC + iPSYCH	20,158 cases; 30,031 controls	1
**ADHD**	PGC + iPSYCH	20,183 cases; 35,191 controls	2
**TD**	PGC	4,642 cases; 8,696 controls	Unpublished (manuscript in preparation)
**OCD**	PGC	2,936 cases; 7,285 controls	3
**ANX**	ANGST	5,761 cases; 11,765 controls	4
**MDD**	PGC	59,851 cases; 113,154 controls	5
**BD**	PGC	11,974 cases; 51,792 controls	6
**SCZ**	PGC	33,640 cases; 43,456 controls	7
**ADHD traits**	EAGLE	17,666	8
**Depressive traits**	SSGAC	180,866	9
**General cognition**	COGENT	35,298	10

ADHD: attention-deficit hyperactivity disorder; ASD: autism spectrum disorder; BD: bipolar disorder; MDD: major depressive disorder; SCZ: schizophrenia; OCD: obsessive-compulsive disorder; TD: Tic disorders; ANX: anxiety disorders; PGC: Psychiatric Genomics Consortium; iPSYCH: Lundbeck Foundation Initiative for Integrative Psychiatric Research; ANGST: Anxiety NeuroGenetics Study; EAGLE: EArly Genetics and Lifecourse Epidemiology Consortium; SSGAC: Social Science Genetic Association Consortium; COGENT: Cognitive Genomics Consortium.

Please note: the power of the discovery GWAS (and hence polygenic risk scores derived from these GWAS) is variable, depending on sample size. The highest power comes from GWAS of depressive traits, MDD, SCZ, ADHD, ASD, BD, with more modest power for scores based on general cognition, ADHD traits, ANX, TD, and OCD.

© 2018 American Medical Association. All rights reserved.

eTable 3. Probandwise Concordances

Disorder	N MZ Concordant Pairs	N MZ Discordant Pairs	N DZ Concordant Pairs	N DZ Discordant Pairs	MZ Probandwise Concordance	DZ Probandwise Concordance
**ASD**	14	35	11	202	.44	.10
**ADHD**	73	97	84	597	.60	.22
**ID**	16	11	7	137	.74	.09
**TD**	5	24	1	76	.29	.03
**OCD**	1	34	1	83	.02	.02
**ANX**	21	100	17	364	.30	.09
**MDD**	21	101	15	298	.29	.09

ASD: autism spectrum disorders; ADHD: attention-deficit/hyperactivity disorder; ID: intellectual disability; TD: Tic disorders; OCD: obsessive-compulsive disorder; ANX: anxiety disorders; MDD: major depressive disorder.

© 2018 American Medical Association. All rights reserved.

eTable 4. Twin Model Fit Statistics for Different Models Comparing the Associations Between Clinical Diagnosis and Continuous Traits

**ASD & ATAC-ASD (9/12)**								
**Model**	**-2LL**	**Parameters**	**df**	**BIC**	**Comparison**	**$\Delta\chi^2$ ***	**Δdf ***	**p ***
**Saturated**	75599.64	25	55601	-454906.04	-----	-----	-----	-----
**ADE-s**	75751.93	14	55614	-454877.79	Saturated	152.29	13	<.001
**ADE**	75815.85	12	55616	-454832.95	ADE-s	63.92	2	<.001
AE-s	***75751.93***	***11***	***55617***	***-454906.47***	***ADE-s***	***0.00***	***3***	***1.00***
**AE**	75959.83	9	55619	-454717.60	ADE-s	207.90	5	<.001
**E**	79055.58	6	55622	-451650.47	ADE-s	3303.65	8	<.001
**ADHD & ATAC-ADHD (9/12)**								
**Model**	**-2LL**	**Parameters**	**df**	**BIC**	**Comparison**	**$\Delta\chi^2$ ***	**Δdf ***	**p ***
**Saturated**	80878.12	25	55580	-449427.20	-----	-----	-----	-----
**ADE-s**	80976.35	14	55593	-449453.00	Saturated	98.23	13	<.001
**ADE**	81013.81	12	55595	-449434.62	ADE-s	37.47	2	<.001
AE-s	***80976.35***	***11***	***55596***	***-449481.63***	***ADE-s***	***0.00***	***3***	***1.00***
**AE**	81173.31	9	55598	-449303.75	ADE-s	196.96	5	<.001
**E**	84193.10	6	55601	-446312.58	ADE-s	3216.75	8	<.001
**ID & ATAC-Learning (9/12)**								
**Model**	**-2LL**	**Parameters**	**df**	**BIC**	**Comparison**	**$\Delta\chi^2$ ***	**Δdf ***	**p ***
**Saturated**	75699.50	25	55625	-455035.17	-----	-----	-----	-----
**ADE-s**	75740.83	14	55638	-455117.88	Saturated	41.33	13	<.001
**ADE**	75808.44	12	55640	-455069.35	ADE-s	67.61	2	<.001
AE-s	***75743.35***	***11***	***55641***	***-455143.98***	***ADE-s***	***2.52***	***3***	***.47***
**AE**	76333.75	9	55643	-454572.66	ADE-s	592.93	5	<.001
**E**	78525.53	6	55646	-452409.51	ADE-s	2784.70	8	<.001

© 2018 American Medical Association. All rights reserved.

TD & ATAC-Tics (9/12)								
**Model**	**-2LL**	**Parameters**	**df**	**BIC**	**Comparison**	$\Delta\chi^2$ *	Δdf *	p *
**Saturated**	78122.01	25	55612	-452488.63	-----	-----	-----	-----
**ADE-s**	78390.99	14	55625	-452343.68	Saturated	268.98	13	<.001
**ADE**	78404.15	12	55627	-452349.60	ADE-s	13.16	2	<.01
**AE-s**	78392.69	11	55628	-452370.60	ADE-s	1.70	3	.64
**AE**	78495.12	9	55630	-452287.26	ADE-s	104.13	5	<.001
**E**	79305.99	6	55633	-451505.01	ADE-s	915.00	8	<.001
Drop s for Tic Disorder	***78392.75***	***10***	***55629***	***-452380.09***	***ADE-s***	***1.76***	***4***	***.78***
**ANX & SCARED (9)**								
**Model**	**-2LL**	**Parameters**	**df**	**BIC**	**Comparison**	$\Delta\chi^2$ *	Δdf *	p *
**Saturated**	47327.92	25	43620	-368863.48	-----	-----	-----	-----
**ADE-s**	47410.78	14	43633	-368904.66	Saturated	82.86	13	<.001
**ACE**	47408.14	12	43635	-368926.37	Saturated	80.23	15	<.001
**AE**	47418.05	9	43638	-368945.08	ACE	9.91	3	<.05
**CE**	47723.29	9	43638	-368639.85	ACE	315.15	3	<.001
**E**	49506.44	6	43641	-366885.32	ACE	2098.30	6	<.001
Drop C for Anxiety, drop rC and rE	***47408.35***	***10***	***43637***	***-368945.25***	***ACE***	***0.21***	***2***	***.90***
**ANX & SDQ-E-parent (15)**								
**Model**	**-2LL**	**Parameters**	**df**	**BIC**	**Comparison**	$\Delta\chi^2$ *	Δdf *	p *
**Saturated**	25775.16	25	35484	-312788.24	-----	-----	-----	-----
**ADE-s**	25812.20	14	35497	-312875.23	Saturated	37.04	13	<.001
**ADE**	25814.47	12	35499	-312892.05	ADE-s	2.27	2	.32
**AE-s**	25812.23	11	35500	-312903.83	ADE-s	0.03	3	1.00
AE	***25816.54***	***9***	***35502***	***-312918.60***	***ADE-s***	***4.34***	***5***	***.50***
**E**	26300.47	6	35505	-312463.29	ADE-s	488.27	8	<.001

© 2018 American Medical Association. All rights reserved.

ANX & SDQ-E-self (15)								
**Model**	**-2LL**	**Parameters**	**df**	**BIC**	**Comparison**	$\Delta\chi^2$ *	Δdf *	p *
**Saturated**	27457.93	25	35971	-44484.07	-----	-----	-----	-----
**ADE-s**	27490.09	14	35984	-44477.91	Saturated	32.16	13	<.01
**ADE**	27492.05	12	35986	-44479.95	ADE-s	1.96	2	.38
**AE-s**	27490.16	11	35987	-44483.84	ADE-s	0.06	3	1.00
**AE**	27497.58	9	35989	-44480.42	ADE-s	7.48	5	.19
**E**	27915.96	6	35992	-44068.04	ADE-s	425.86	8	<.001
Drop rE	***27499.40***	***8***	***35990***	***-44480.60***	***ADE-s***	***9.30***	***6***	***.16***

ANX & ABCL-anx (18)								
**Model**	**-2LL**	**Parameters**	**df**	**BIC**	**Comparison**	$\Delta\chi^2$ *	Δdf *	p *
**Saturated**	18751.80	25	32981	-295929.73	-----	-----	-----	-----
**ADE-s**	18766.53	14	32994	-296039.04	Saturated	14.73	13	.32
**ACE**	18772.13	12	32996	-296052.52	Saturated	20.33	15	.16
AE	***18774.04***	***9***	***32999***	***-296079.24***	***ACE***	***1.91***	***3***	***.59***
**CE**	18850.35	9	32999	-296002.92	ACE	78.22	3	<.001
**E**	19348.66	6	33002	-295533.24	ACE	576.52	6	<.001

ANX & SCARED (18)								
**Model**	**-2LL**	**Parameters**	**df**	**BIC**	**Comparison**	$\Delta\chi^2$ *	Δdf *	p *
**Saturated**	20701.85	25	33622	-300095.65	-----	-----	-----	-----
**ADE-s**	20748.94	14	33635	-300172.60	Saturated	47.08	13	<.001
**ADE**	20754.03	12	33637	-300186.60	ADE-s	5.09	2	.08
AE-s	***20748.94***	***11***	***33638***	***-300201.23***	***ADE-s***	***0.00***	***3***	***1.00***
**AE**	20764.16	9	33640	-300205.09	ADE-s	15.22	5	<.05
**E**	21116.41	6	33643	-299881.46	ADE-s	367.47	8	<.001

© 2018 American Medical Association. All rights reserved.

MDD & SMFQ (9)								
Model	-2LL	Parameters	df	BIC	Comparison	$\Delta\chi^2$ *	Δdf *	p *
Saturated	47898.84	25	43694	-368998.61	-----	-----	-----	-----
ADE-s	48060.39	14	43707	-368961.09	Saturated	161.55	13	<.001
ACE	48056.75	12	43709	-368983.82	Saturated	157.90	15	<.001
AE	***48061.16***	***9***	***43712***	***-369008.04***	***ACE***	***4.41***	***3***	***.22***
CE	48236.30	9	43712	-368832.89	ACE	179.56	3	<.001
E	49346.94	6	43715	-367750.87	ACE	1290.20	6	<.001
Drop rE	48063.41	8	43713	-369015.32	ACE	6.67	4	.15
**MDD & SDQ-E-parent (15)**								
Model	-2LL	Parameters	df	BIC	Comparison	$\Delta\chi^2$ *	Δdf *	p *
Saturated	25299.73	25	35484	-313263.67	-----	-----	-----	-----
ADE-s	25334.64	14	35497	-313352.79	Saturated	34.92	13	<.01
ACE	25337.37	12	35499	-313369.15	Saturated	37.64	15	<.01
AE	***25337.37***	***9***	***35502***	***-313397.77***	***ACE***	***0.00***	***3***	***1.00***
CE	25424.06	9	35502	-313311.08	ACE	86.69	3	<001
E	25835.23	6	35505	-312928.53	ACE	497.86	6	<.001
**MDD & SDQ-E-self (15)**								
Model	-2LL	Parameters	df	BIC	Comparison	$\Delta\chi^2$ *	Δdf *	p *
Saturated	26962.02	25	35971	-316247.99	-----	-----	-----	-----
ADE-s	26997.22	14	35984	-316336.83	Saturated	35.20	13	<.01
ACE	27001.82	12	35986	-316351.31	Saturated	39.81	15	<.001
AE	***27001.95***	***9***	***35989***	***-316379.80***	***ACE***	***0.13***	***3***	***.99***
CE	27080.88	9	35989	-316300.87	ACE	79.06	3	<.001

© 2018 American Medical Association. All rights reserved.

**E**	27435.39	6	35992	-315974.99	ACE	433.57	6	<.001

MDD & ABCL-dep (18)								
**Model**	**-2LL**	**Parameters**	**df**	**BIC**	**Comparison**	**Δχ² ***	**Δdf ***	**p ***
**Saturated**	18385.45	25	33036	-296820.85	-----	-----	-----	-----
**ADE-s**	18400.23	14	33049	-296930.11	Saturated	14.78	13	.32
**ACE**	18405.69	12	33051	-296943.73	Saturated	20.24	15	.16
AE	***18405.91***	***9***	***33054***	***-296972.13***	***ACE***	***0.22***	***3***	***.97***
**CE**	18483.42	9	33054	-296894.62	ACE	77.73	3	<.001
**E**	18891.61	6	33057	-296515.06	ACE	485.91	6	<.001
**MDD & CESD (18)**								
**Model**	**-2LL**	**Parameters**	**df**	**BIC**	**Comparison**	**Δχ² ***	**Δdf ***	**p ***
**Saturated**	19615.60	25	33339	-298481.72	-----	-----	-----	-----
**ADE-s**	19646.94	14	33352	-298574.41	Saturated	31.35	13	<.01
**ACE**	19651.22	12	33354	-298589.22	Saturated	35.62	15	<.01
AE	***19651.29***	***9***	***33357***	***-298617.77***	***ACE***	***0.07***	***3***	***1.00***
**CE**	19716.58	9	33357	-298552.47	ACE	65.37	3	<.001
**E**	20012.23	6	33360	-298285.45	ACE	361.01	6	<.001

ASD: autism spectrum disorders; ADHD: attention-deficit/hyperactivity disorder; ID: intellectual disability; TD: Tic disorders; ANX: anxiety disorders; MDD: major depressive disorder

ATAC: Autism-Tics, AD/HD, and other Comorbidities inventory; SCARED: Screen for Anxiety Related Disorders; SDQ-E-parent: parent-rated Strengths and Difficulties Questionnaire, Emotional problems subscale; SDQ-E-self: self-rated Strengths and Difficulties Questionnaire, Emotional problems subscale; ABCL-anx: Adult Behavior

© 2018 American Medical Association. All rights reserved.

Checklist, DSM-IV anxiety subscale; SMFQ: Short Mood and Feelings Questionnaire; ABCL-dep: Adult Behavior Checklist, DSM-IV depression subscale; CESD: Center for Epidemiologic Studies Depression Scale

* $\Delta\chi 2$: difference in -2LL between two models, distributed $\chi 2$; Δdf: difference in degrees of freedom between two models; p: p-value from likelihood-ratio tests.

All ADE-s and ACE models are compared with the saturated model. The column labelled 'Comparison' shows which models are being compared by the likelihood-ratio test.

The best-fitting model is indicated in bold italics in each case.

© 2018 American Medical Association. All rights reserved.

eTable 5 Parameter Estimates From the Best-Fitting Twin Models of the Associations Between Continuous Traits and Clinical Diagnoses

	A	D	C	E	s	rA (% rPH A)	rA^2	rE (% rPH E)	rE^2
**ASD & ATAC-ASD (9/12)**									
ATAC-ASD	.79 (.78-.80)	-----	-----	.21 (.20-.22)	-.09 (-.10/-.08)	.48 (.44-.53)	.23	.27 (.16-.38)	.07
ASD	.84 (.70-.91)	-----	-----	.16 (.09-.30)	-.06 (-.12/.01)	89%		11%	
**ADHD & ATAC-ADHD (9/12)**									
ATAC-ADHD	.76 (.75-.78)	-----	-----	.24 (.22-.25)	-.09 (-.10/-.08)	.56 (.53-.59)	.31	.36 (.28-.45)	.12
ADHD	.89 (.88-.89)	-----	-----	.11 (.11-.12)	-.01 (-.05/.03)	89%		11%	
**ID & ATAC-Learning (9/12)**									
ATAC-Learning	.81 (.80-.82)	-----	-----	.19 (.18-.20)	-.15 (-.16/-.14)	.69 (.64-.73)	.48	.55 (.31-.75)	.30
ID	.94 (.85-.98)	-----	-----	.06 (.02-.15)	-.09 (-.14/-.02)	91%		9%	
**TD & ATAC-Tics (9/12)**									
ATAC-Tics	.56 (.52-.59)	-----	-----	.44 (.41-.48)	-.09 (-.11/-.07)	.61 (.51-.80)	.37	.31 (.18-.44)	.10
TD	.57 (.32-.77)	-----	-----	.43 (.25-.68)	-----	72%		28%	
**ANX & SCARED (9)**									
SCARED	.58 (.52-.64)	-----	.08 (.03-.12)	.34 (.32-.36)	-----	.48 (.37-.63)	.23	-----	-----
ANX	.67 (.43-.76)	-----	.00 (.00-.02)	.33 (.24-.44)	-----	100%		-----	-----
**ANX & SDQ-E-parent (15)**									
SDQ-E-parent	.46 (.42-.49)	-----	-----	.54 (.51-.58)	-----	.46 (.34-.58)	.21	.26 (.11-.40)	.07
ANX	.66 (.55-.76)	-----	-----	.34 (.24-.45)	-----	70%		30%	

© 2018 American Medical Association. All rights reserved.

	A	D	C	E	s	rA (% rPH A)	rA^2	rE (% rPH E)	rE^2
**ANX & SDQ-E-self (15)**									
SDQ-E-self	.42 (.38-.45)	-----	-----	.58 (.55-.62)	-----	.47 (.37-.57)	.22	-----	-----
ANX	.67 (.55-.76)	-----	-----	.33 (.24-.45)	-----	100%		-----	-----
**ANX & ABCL-anx (18)**									
	A	D	C	E	s	rA (% rPH A)	rA^2	rE (% rPH E)	rE^2
ABCL-anx	.57 (.53-.61)	-----	-----	.43 (.39-.47)	-----	.47 (.36-.58)	.22	.29 (.13-.44)	.08
ANX	.66 (.54-.75)	-----	-----	.34 (.25-.46)	-----	72%		28%	
**ANX & SCARED (18)**									
	A	D	C	E	s	rA (% rPH A)	rA^2	rE (% rPH E)	rE^2
SCARED (18)	.58 (.50-.65)	-----	-----	.42 (.35-.50)	-.07 (-.11/-.03)	.57 (.46-.68)	.32	.15 (-.03-.33)	.02
ANX	.74 (.61-.83)	-----	-----	.26 (.17-.39)	-.06 (-.12/.01)				
**MDD & SMFQ (9)**									
	A	D	C	E	s	rA (% rPH A)	rA^2	rE (% rPH E)	rE^2
SMFQ	.56 (.53-.58)	-----	-----	.44 (.42-.47)	-----	.33 (.19-.47)	.11	-----	-----
DEP	.70 (.59-.78)	-----	-----	.30 (.22-.41)	-----	100%		-----	-----
**MDD & SDQ-E-parent (15)**									
	A	D	C	E	s	rA (% rPH A)	rA^2	rE (% rPH E)	rE^2
SDQ-E-parent	.46 (.38-.45)	-----	-----	.54 (.51-.58)	-----	.39 (.27-.51)	.15	.35 (.20.49)	.12
DEP	.68 (.58-.77)	-----	-----	.32 (.23-.42)	-----	60%		40%	
**MDD & SDQ-E-self (15)**									
	A	D	C	E	s	rA (% rPH A)	rA^2	rE (% rPH E)	rE^2
SDQ-E-self	.42 (.38-.45)	-----	-----	.58 (.55-.62)	-----	.33 (.20-.46)	.11	.22 (.07-.37)	.05

© 2018 American Medical Association. All rights reserved.

	A	D	C	E	s	rA (% rPH A)	rA²	rE (% rPH E)	rE²
DEP	.69 (.59-.78)	-----	-----	.31 (.22-.41)	-----	65%		35%	
**MDD & ABCL-dep (18)**									
	A	D	C	E	s	rA (% rPH A)	rA²	rE (% rPH E)	rE²
ABCL-dep	.51 (.47-.55)	-----	-----	.49 (.45-.53)	-----	.53 (.50-.56)	.28	.32 (.26-.45)	.10
DEP	.67 (.64-.76)	-----	-----	.33 (.30-.36)	-----	70%		30%	
**MDD & CESD (18)**									
	A	D	C	E	s	rA (% rPH A)	rA²	rE (% rPH E)	rE²
CESD	.44 (.39-.49)	-----	-----	.56 (.51-.61)	-----	.58 (.52-.63)	.34	.30 (.11-.41)	.09
DEP	.68 (.63-.73)	-----	-----	.32 (.27-.37)	-----	71%		29%	

ASD: autism spectrum disorders; ADHD: attention-deficit/hyperactivity disorder; ID: intellectual disability; TD: Tic disorders; ANX: anxiety disorders; MDD: major depressive disorder

ATAC: Autism-Tics, AD/HD, and other Comorbidities inventory; SCARED: Screen for Anxiety Related Disorders; SDQ-E-parent: parent-rated Strengths and Difficulties Questionnaire, Emotional problems subscale; SDQ-E-self: self-rated Strengths and Difficulties Questionnaire, Emotional problems subscale; ABCL-anx: Adult Behavior Checklist, DSM-IV anxiety subscale; SMFQ: Short Mood and Feelings Questionnaire; ABCL-dep: Adult Behavior Checklist, DSM-IV depression subscale; CESD: Center for Epidemiologic Studies Depression Scale

A: additive genetic influences; D: non-additive genetic influences; C: shared environmental influences; E: nonshared environmental influences; s: sibling interaction effects; rA: additive genetic correlation; rD: non-additive genetic correlation; rC: shared environmental correlation; rE: nonshared environmental correlation; % rPH A: percentage of the phenotypic correlation explained by A; % rPH C: percentage of the phenotypic correlation explained by C; % rPH D: percentage of the phenotypic correlation explained by D; % rPH E: percentage of the phenotypic correlation explained by E; rA²: squared genetic correlation, which gives the proportion of genetic variance shared between two phenotypes; rE²: squared nonshared environmental correlation, which gives the proportion of nonshared environmental variance shared between two phenotypes

© 2018 American Medical Association. All rights reserved.

eTable 6. Twin Fit Statistics for Diagnostic Subdomains

ASD & ATAC-Social (9/12)								
**Model**	**-2LL**	**Parameters**	**df**	**BIC**	**Comparison**	**$\Delta\chi^2$ ***	**Δdf ***	**p ***
**Saturated**	77288.36	25	55606	-453265.02	-----	-----	-----	-----
**ADE-s**	77468.82	14	55619	-453208.60	Saturated	180.45	13	<.001
**ADE**	77540.54	12	55621	-453155.97	ADE-s	71.71	2	<.001
AE-s	***77468.82***	***11***	***55622***	***-453237.23***	***ADE-s***	***0.00***	***3***	***1.00***
**AE**	77642.00	9	55624	-453083.13	ADE-s	173.18	5	<.001
**E**	79555.07	6	55627	-451198.68	ADE-s	2086.25	8	<.001
**ASD & ATAC-Language (9/12)**								
**Model**	**-2LL**	**Parameters**	**df**	**BIC**	**Comparison**	**$\Delta\chi^2$ ***	**Δdf ***	**p ***
**Saturated**	76053.65	25	55614	-454576.07	-----	-----	-----	-----
**ADE-s**	76085.11	14	55627	-454668.64	Saturated	31.46	13	<.01
**ADE**	76089.83	12	55629	-454683.00	ADE-s	4.72	2	.09
AE-s	***76090.69***	***11***	***55630***	***-454691.69***	***ADE-s***	***5.58***	***3***	***.13***
**AE**	76261.76	9	55632	-454529.70	ADE-s	176.64	5	<.001
**E**	79208.43	6	55635	-451621.65	ADE-s	3123.32	8	<.001
**ASD & ATAC-Flexibility (9/12)**								
**Model**	**-2LL**	**Parameters**	**df**	**BIC**	**Comparison**	**$\Delta\chi^2$ ***	**Δdf ***	**p ***
**Saturated**	77740.44	25	55610	-452851.11	-----	-----	-----	-----
**ADE-s**	78021.60	14	55623	-452693.99	Saturated	281.16	13	<.001
**ADE**	78129.28	12	55625	-452605.39	ADE-s	107.68	2	<.001
AE-s	***78021.60***	***11***	***55626***	***-452722.61***	***ADE-s***	***0.00***	***3***	***1.00***
**AE**	78336.40	9	55628	-452426.90	ADE-s	314.79	5	<.001
**E**	79958.72	6	55631	-450833.20	ADE-s	1937.11	8	<.001

© 2018 American Medical Association. All rights reserved.

ADHD & ATAC-Hyperactivity/Impulsivity (9/12)								
**Model**	**-2LL**	**Parameters**	**df**	**BIC**	**Comparison**	$\Delta\chi^2$ *	Δdf *	p *
**Saturated**	81867.54	25	55597	-448599.97	-----	-----	-----	-----
**ADE-s**	81948.02	14	55610	-448643.53	Saturated	80.48	13	<.001
**ACE**	82310.79	12	55612	-448299.85	Saturated	443.25	15	<.001
**ADE**	82004.70	12	55612	-448605.93	ADE-s	56.68	2	<.001
AE-s	***81948.11***	***11***	***55613***	***-448672.07***	***ADE-s***	***0.09***	***3***	***.99***
**AE**	82310.79	9	55615	-448328.47	ADE-s	362.76	5	<.001
**E**	85101.86	6	55618	-445566.02	ADE-s	3153.84	8	<.001
**ADHD & ATAC-Inattention (9/12)**								
**Model**	**-2LL**	**Parameters**	**df**	**BIC**	**Comparison**	$\Delta\chi^2$ *	Δdf *	p *
**Saturated**	81840.90	25	55595	-448607.53	-----	-----	-----	-----
**ADE-s**	81916.85	14	55608	-448655.62	Saturated	75.94	13	<.001
**ACE**	82231.49	12	55610	-448360.06	Saturated	390.59	15	<.001
**ADE**	81980.76	12	55610	-448610.79	ADE-s	63.91	2	<.001
AE-s	***81916.85***	***11***	***55611***	***-448684.39***	***ADE-s***	***0.00***	***3***	***1.00***
**AE**	82231.49	9	55613	-448388.68	ADE-s	314.65	5	<.001
**E**	84112.87	6	55616	-446535.92	ADE-s	2196.03	8	<.001

* $\Delta\chi2$: difference in -2LL between two models, distributed $\chi2$; Δdf: difference in degrees of freedom between two models; p: p-value from likelihood-ratio tests

All ADE-s and ACE models are compared with the saturated model. The column labelled 'Comparison' shows which models are being compared by the likelihood-ratio test.

The best-fitting model is indicated in bold italics in each case.

© 2018 American Medical Association. All rights reserved.

eTable 7: Twin Model Estimates for Diagnostic Subdomains

ASD & ATAC-Social (9/12)									
	A	D	C	E	s	rA (% rPH A)	rD (% rPH D)	rC (% rPH C)	rE (% rPH E)
ATAC-Social	.71 (.69-.73)	-----	-----	.29 (.27-.31)	-.09 (-.11/-.08)	.48 (.43-.55)	-----	-----	.31 (.19-.43)
ASD	.83 (.69-.91)	-----	-----	.17 (.09-.31)	-.05 (-.11/.02)	84%	-----	-----	16%
**ASD & ATAC-Language (9/12)**									
	A	D	C	E	s	rA (% rPH A)	rD (% rPH D)	rC (% rPH C)	rE (% rPH E)
ATAC-Language	.75 (.74-.77)	-----	-----	.25 (.23-.26)	-.08 (-.09/-.07)	.43 (.39-.47)	-----	-----	.20 (.14-.32)
ASD	.88 (.79-.92)	-----	-----	.12 (.08-.21)	-.08 (-.14/-.02)	91%	-----	-----	9%
**ASD & ATAC-Flexibility (9/12)**									
	A	D	C	E	s	rA (% rPH A)	rD (% rPH D)	rC (% rPH C)	rE (% rPH E)
ATAC-Flexibility	.74 (.72-.76)	-----	-----	.26 (.24-.28)	-.12 (-.14/-.11)	.47 (.46-.51)	-----	-----	.23 (.10-.35)
ASD	.85 (.72-.91)	-----	-----	.15 (.09-.28)	-.04 (-.10/.03)	89%	-----	-----	11%
**ADHD & ATAC-Hyperactivity/Impulsivity (9/12)**									
	A	D	C	E	s	rA (% rPH A)	rD (% rPH D)	rC (% rPH C)	rE (% rPH E)
ATAC-HYP/IMP	.79 (.77-.80)	-----	-----	.21 (.20-.23)	-.12 (-.13/-.11)	.49 (.46-.52)	-----	-----	.26 (.17-.35)
ADHD	.89 (.84-.93)	-----	-----	.11 (.07 -.16)	.00 (-.05/.04)	91%	-----	-----	9%
**ADHD & ATAC-Inattention (9/12)**									
	A	D	C	E	s	rA (% rPH A)	rD (% rPH D)	rC (% rPH C)	rE (% rPH E)
ATAC-Inattention	.73 (.71-.74)	-----	-----	.27 (.26-.29)	-.13 (-.14/-.11)	.57 (.54-.60)	-----	-----	.41 (.32-.50)
ADHD	.89 (.83-.93)	-----	-----	.11 (.07-.17)	-.01 (-.05/.04)	86%	-----	-----	14%

ASD: autism spectrum disorders; ADHD: attention-deficit/hyperactivity disorder; A-TAC: Autism-Tics, AD/HD, and other Comorbidities inventory; HYP/IMP: Hyperactivity/Impulsivity subscale.

A: additive genetic influences; D: non-additive genetic influences; C: shared environmental influences; E: nonshared environmental influences; s: sibling interaction effects; rA: additive genetic correlation; rD: non-additive genetic correlation; rC: shared environmental correlation; rE: nonshared environmental correlation; % rPH A:

© 2018 American Medical Association. All rights reserved.

percentage of the phenotypic correlation explained by A; % rPH C: percentage of the phenotypic correlation explained by C; % rPH D: percentage of the phenotypic correlation explained by D; % rPH E: percentage of the phenotypic correlation explained by E.

© 2018 American Medical Association. All rights reserved.

eTable 8. Association of Polygenic Risk Scores for ADHD and ASD With Traits of Diagnostic Subdomains

PRS	Outcome	Full sample					Excluding those with ICD diagnoses				
		N	Beta	SE	P	R^2	N	Beta	SE	P	R^2
**ASD**	Social	13397	0.011	0.006	0.042*	3.9E-04	13256	0.009	0.005	0.055	3.2E-04
	Language	13398	0.011	0.006	0.047*	4.2E-04	13256	0.008	0.005	0.15	2.1E-04
	Flexibility	13397	0.022	0.005	6.9E-06***	1.5E-03	13255	0.020	0.005	8.7E-05***	1.4E-03
**ADHD**	Hyperactivity/ Impulsivity	13395	0.139	0.015	1.9E-19***	7.7E-03	12957	0.109	0.014	2.5E-14***	5.9E-03
	Inattention	13394	0.129	0.016	2.9E-15***	6.0E-03	12957	0.096	0.015	2.6E-10***	4.1E-03

*p<0.05, **p<0.01, ***p<0.001. ASD: autism spectrum disorders; ADHD: attention-deficit/hyperactivity disorder.

© 2018 American Medical Association. All rights reserved.

eFigure. Results of Sensitivity Analyses

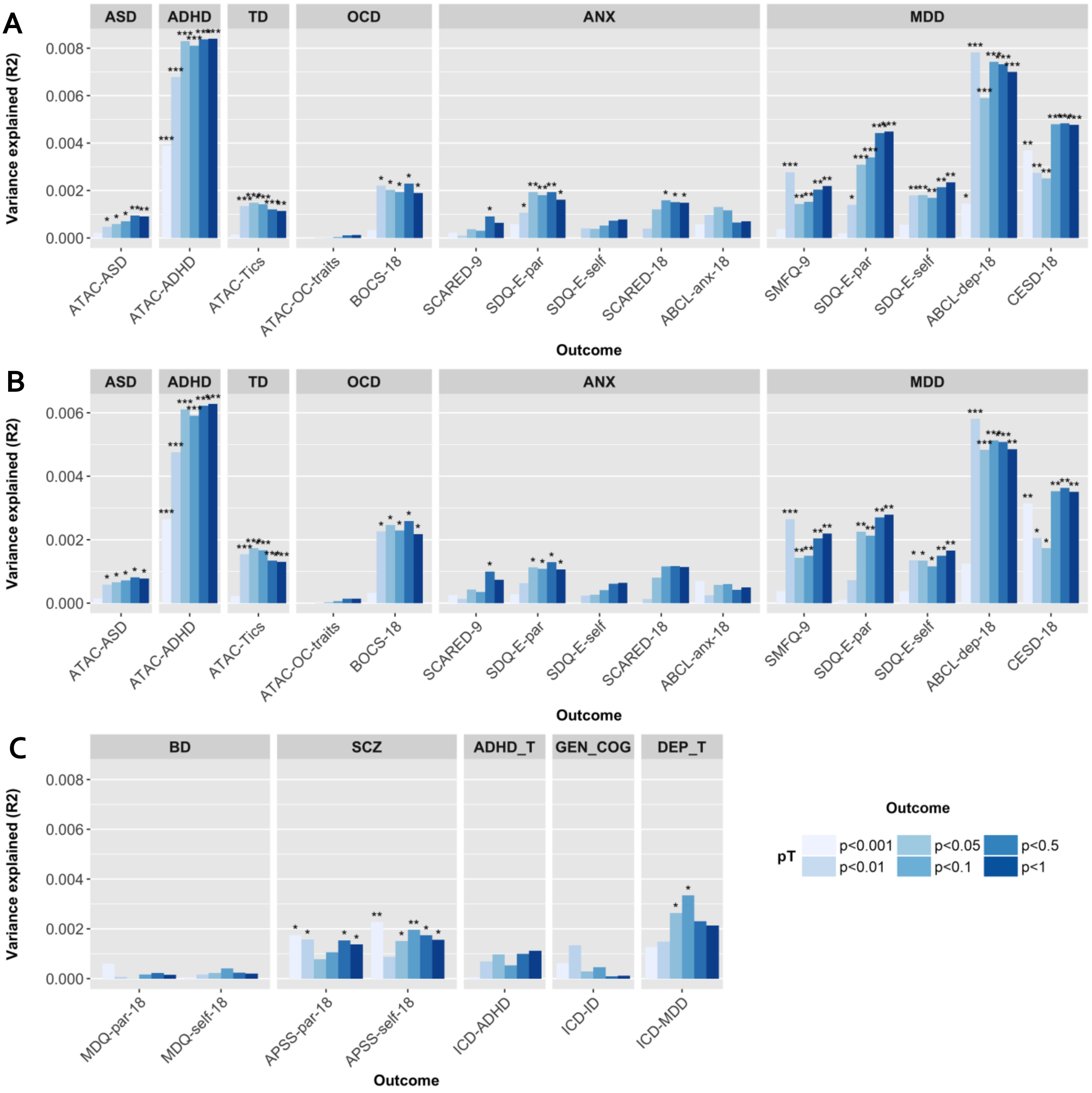

Results of sensitivity analyses using polygenic risk scores (PRS) derived based on variable p-value thresholds (pT), for: a) Associations between PRS with continuous psychiatric outcomes in the full sample; b) PRS associations with the same outcomes as shown in a), after excluding individuals with the relevant ICD diagnosis from each analysis; c) PRS associations with outcomes in the full sample, for late-onset phenotypes, where individuals with ICD diagnoses could not be excluded and for analyses of binary ICD diagnosis outcomes.

Discovery GWAS samples (based on case-control and quantitative trait GWAS) used to derive PRS are listed above each plot. Asterisks indicate significance after false discovery rate (FDR) correction: *$p_{FDR}<0.05$, **$p_{FDR}<0.01$, ***$p_{FDR}<0.001$. ASD: autism spectrum disorder; ADHD: attention-deficit hyperactivity disorder; TD: Tic disorders; OCD: obsessive-compulsive disorder; ANX: anxiety disorders; MDD: major depressive disorder; BD: bipolar disorder; SCZ: schizophrenia; ADHD_T: ADHD traits; GEN_COG: General cognitive ability; ID: intellectual disability; DEP_T: Depressive traits.

© 2018 American Medical Association. All rights reserved.
